# Diagnostic Potential of IgG and IgA Responses to *Mycobacterium tuberculosis* Antigens for Discrimination among Active Tuberculosis, Latent Tuberculosis Infection, and Non-Infected Individuals

**DOI:** 10.3390/microorganisms8070979

**Published:** 2020-06-30

**Authors:** Ji Yeon Lee, Byoung-Jun Kim, Hyeon-Kyoung Koo, Junghyun Kim, Jee-min Kim, Yoon-Hoh Kook, Bum-Joon Kim

**Affiliations:** 1 Division of Pulmonary and Critical Care Medicine, Department of Internal Medicine, National Medical Center, Seoul 04564, Korea; fulgeo@nmc.or.kr (J.Y.L.); splendor329@hanmail.net (J.K.); everflying@hanmail.net (J.-m.K.); 2 Department of Biomedical Sciences, Microbiology and Immunology and Liver Research Institute, College of Medicine, Seoul National University, Seoul 03080, Korea; arukas22@snu.ac.kr; 3 Division of Pulmonary and Critical Care Medicine, Department of Internal Medicine, Ilsan Paik Hospital, Inje University College of Medicine, Ilsan 10380, Korea; gusrud.koo@gmail.com

**Keywords:** tuberculosis, serology, diagnosis, biomarkers

## Abstract

Tuberculosis remains a major public health problem. Conventional tests are inadequate to distinguish between active tuberculosis (ATB) and latent tuberculosis infection (LTBI). We measured antibody responses to *Mycobacterium tuberculosis* antigens (*Mycobacterium tuberculosis* chorismate mutase (TBCM), antigen 85B (Ag85B), early secreted antigen-6 (ESAT-6), and culture filtrate protein-10 (CFP-10) in ATB, LTBI, and non-infected (NI) individuals. Serum immunoglobulin G (IgG) and immunoglobulin A (IgA) levels were measured and the QuantiFERON-TB Gold In-Tube assay was used to diagnose LTBI. IgG levels against TBCM were significantly higher in LTBI than NI subjects. IgG and IgA levels against Ag85B and IgG levels against CFP-10 were significantly higher in ATB, followed by LTBI, and then NI. When the ATB group was subdivided, IgG levels against Ag85B and CFP-10 were significantly higher in each subgroup compared with those in LTBI and NI groups. Positive correlation trends between interferon-gamma and IgG levels against Ag85B, TBCM, and CFP-10 and IgA levels against Ag85B in LTBI and NI subjects were observed. Age- and sex-adjusted models showed that IgG against TBCM and CFP-10 was independently related to LTBI diagnosis, and IgG against Ag85B was independently related to the diagnosis of ATB and could distinguish between LTBI and ATB. Overall, IgG antibody responses to TBCM, Ag85B, and CFP-10 can discriminate among ATB, LTBI, and NI groups.

## 1. Introduction

Tuberculosis (TB) remains a major public health problem. In 2018, 7.0 million new cases of TB were reported worldwide, as well as an estimated 1.2 million TB deaths among human immunodeficiency virus (HIV)-negative people and an additional 251,000 deaths among HIV-positive people [1]. In South Korea, 26,433 new TB cases (51.5 cases per 100,000 people) were reported in 2018 [2]. Since the establishment of the national TB control program in 1962 [3], the number of reported pulmonary TB patients in South Korea has rapidly declined, from 101 per 100,000 people in 1995 to 79 in 2002. However, since then, the decline has slowed due to an increase in the elderly population and the number of immunocompromised patients, as well as frequent outbreaks of TB in group facilities [4].

The rapid diagnosis and treatment of TB is essential to contain the disease at an early stage and to lower its prevalence. In addition, diagnosis and treatment of latent tuberculosis infection (LTBI) as well as active tuberculosis (ATB) are required for effective TB control. Latency and active disease are components of the dynamic spectrum of TB [5]. As latent TB bacilli could reactivate later to cause active TB, diagnosis and treatment of LTBI is also important.

The diagnosis of TB is confirmed by a culture of *Mycobacterium tuberculosis* (*Mtb*) but is typically delayed because it takes approximately 2–8 weeks to receive the results [6]. Moreover, there are no diagnostic gold standards for LTBI, and all existing tests for LTBI are indirect approaches that provide immunological evidence of host sensitization to TB antigens [7]. Two tests currently used to diagnose LTBI are the tuberculin skin test and the blood interferon-gamma release assay, both of which do not distinguish between ATB and LTBI [7]. Current tests for LTBI use indirect methods to test for the presence of an immune response to *Mtb*, rather than a direct method to detect a small number of *Mtb* organisms in the body. Therefore, the test result could be positive for cured TB patients.

Recent studies have suggested the utility of antibody responses to TB antigens for the diagnosis of TB. Legesse et al. reported that IgA levels for early secreted antigen-6 (ESAT-6), culture filtrate protein-10 (CFP-10), and Rv2031 could be used to distinguish among patients with pulmonary TB, patients with LTBI, and non-infected (NI) individuals [8]. Other studies have also reported that antibody levels against *Mtb* components are markers for bacterial load and are associated with disease risk [9].

ESAT-6 and CFP-10 are TB-specific secreted proteins encoded by the RD1 gene of *Mtb* [10]; they are not present in Bacillus Calmette-Guérin strains and in most other non-tuberculosis mycobacterial species [11]. ESAT-6 has been identified as a promising component for vaccine development with regard to human T cell recognition and protective efficacy [12,13].

Similarly, antigen 85B (Ag85B) has been investigated as a major antigen in candidate vaccines due to its adaptability and ability to induce CD4 and CD8 T lymphocyte responses in a wide range of vertebrate hosts [13]. Ag85B is a secreted protein of TB and has been considered a potential drug target for TB treatment due to its enzymatic activity as a mycolyl transferase and its importance in the construction of the mycobacterial envelope [14]. Ag85B is highly immunogenic, resulting in specific humoral and cell-mediated immune responses in both LTBI and ATB patients, and has been shown to induce partial protection in murine models of infection [15,16].

In recent studies to explore potential targets for new anti-tubercular agents, chorismate mutase, found in *Mtb*, has been shown to be a key regulator of amino acid biosynthesis [17]. *Mycobacterium tuberculosis* chorismate mutase (TBCM) converts chorismate to prephenate to form the essential amino acids phenylalanine and tyrosine in the shikimate biosynthetic pathway, which plays an important role in the survival and pathophysiology of *Mtb*. Therefore, TBCM is considered a promising target for potential anti-tubercular agents that inhibit this pathway [17]. To date, no studies have investigated antibody responses to TBCM in TB patients.

The aim of the present study was to evaluate diagnostic immune markers using promising immune-dominant TB antigens, which are being studied for the development of TB vaccines and therapeutic drugs, and a recently noted novel antigen, in an intermediate TB-burden setting. We analyzed the immunoglobulin G (IgG) and immunoglobulin A (IgA) responses to TBCM, Ag85B, ESAT-6, and CFP-10 in the sera of patients with ATB and LTBI, as well as in NI individuals.

## 2. Materials and Methods

### 2.1. Participants and Clinical Samples

The inclusion criteria allowed for men and women over 19 years of age, who were diagnosed with ATB or were targeted for the examination of LTBI (contact with persons with ATB, health-care workers, nursery workers, or welfare facility workers) at the National Medical Center, Seoul, Republic of Korea. Individuals under the age of 19 were excluded. Five milliliters of peripheral blood was collected in plain tubes and centrifuged to separate the serum within 24 h of collection. Subsequently, the tubes were stored at −70°C until further analyses.

ATB was confirmed in patients with sputum or bronchoscopy specimens that were positive for either mycobacterial culture or the TB nucleic acid amplification test. The subjects selected for LTBI examination were tested with the QuantiFERON-TB Gold In-Tube (QFT-GIT) assay. Patients with a positive test result in the absence of clinical symptoms and chest radiographic abnormalities were classified as LTBI, and those with a negative test result were classified as NI. Clinical and laboratory data were collected at the baseline examination and at the time of a usual hospital visit.

### 2.2. Preparation of Mycobacterium tuberculosis (Mtb) Antigens

Recombinant Ag85B, CFP10, ESAT-6, and TBCM proteins were purified from *Escherichia coli* as previously described [18,19]. Briefly, *E. coli* BL21 strains (RBC Bioscience, Taipei City, Taiwan) were transformed with pET28a-Ag85B, pET28a-CFP10, pET28a-ESAT-6, or pET28a-TBCM for the expression and purification of each fusion protein. Protein expression was induced by adding 0.4 mM isopropyl β-D-thiogalactoside (IPTG, Duchefa Biochemie, Haarlem, The Netherlands). Cultured bacterial cells were disrupted by sonication (10 min, 4 °C), and the resultant lysates were centrifuged (1600× *g*, 20 min, 4 °C). The pellets containing each protein (Ag85B, CFP-10, ESAT-6, or TBCM) were resuspended in binding buffer containing 4 M urea (Sigma Aldrich, St. Louis, MO, USA). Each protein was purified with Ni-NTA His binding resin (Merck, Darmstadt, Germany) and eluted with elution buffer (300 mM NaCl, 50 mM sodium phosphate buffer, and 250 mM imidazole) containing 4 M urea. Purified proteins were dialyzed serially against the elution buffer to remove imidazole, urea, and residual salts.

### 2.3. Antibody Enzyme-Linked Immunosorbent Assay (ELISA)

Serum levels of antibody isotypes IgA and IgG against TBCM, ESAT-6, Ag85B, and CFP-10 were measured by enzyme-linked immunosorbent assay (ELISA). Corning 96-well Enzyme Immunoassay/Radio Immunoassay (EIA/RIA) plates (Corning Inc., Kennebunk, ME, USA) were coated with TBCM (5 μg/mL), ESAT-6 (5 μg/mL), Ag85B (5 μg/mL), or CFP-10 (5 μg/mL), diluted in 0.05 M carbonate-bicarbonate coating buffer and incubated overnight at 4 °C. Plates were washed three times with PBST (phosphate-buffered saline (PBS) containing 0.05% tween 20) and blocked with PBS containing 5% bovine serum albumin (BSA) for 1 h at room temperature (RT). After washing, 100 μL of sample diluted 1:10 in PBS was added to each well, and plates were incubated at RT for 2 h. After washing, 100 μL of anti-human IgG or IgA HRP-conjugated secondary antibody (IgG: Promega, W4038, Madison, WI, USA; IgA: Invitrogen, PA1-74395, Rockford, IL, USA) diluted at 1:500 in 5% BSA was added to each well of the respective plates. Plates were incubated at RT for 1 h, and after washing, 100 μL of 3, 3’5, 5’-tetramethylbenzidine substrate reagent (BD OptEIA substrate; BD Biosciences, San Diego, CA, USA) was added to each well. After 10 min, the reaction was stopped with 1 N sulfuric acid (50 μL) and analyzed at 450 nm. Optical density (OD) values were used for analysis.

### 2.4. Data Analysis

Antibody levels were presented as OD values, and one-way ANOVA with Bonferroni’s multiple comparisons was used to compare antibody responses among ATB, LTBI, and NI groups. Correlations between the OD values of IgG or IgA and the level of interferon-gamma (IFN-γ) were assessed using Pearson’s correlation coefficient. *p* values less than 0.05 were considered statistically significant. SPSS version 17.0 (SPSS Institute, Inc., Chicago, IL, USA) and GraphPad Prism version 5.00 for Windows (GraphPad Software, La Jolle, CA, USA, http://www.graphpad.com) were used for data analyses. For multivariate analysis, logistic regression with the backward elimination method was performed to predict LTBI or ATB. The area under the curve (AUC) of the receiver operating characteristics (ROC) curve was calculated to compare the predictive power of each model using the ROCR package in R (version 3.6.0). To generate the decision tree for the prediction of diseases, the tree package was used. Tree is a nonparametric statistical procedure containing classification using a set of if-then-else logical conditions to assign unknown features to predefined categories. Algorithms to construct trees work from the top down, by choosing a variable at each step that best separates the set of items. Training and test sets were divided at a 7:3 ratio for cross validation, and the number of pruning nodes was selected by K-fold cross validation.

### 2.5. Ethics Statement

All subjects provided written, informed consent for inclusion before they participated in the study. The study was conducted in accordance with the Declaration of Helsinki, and the protocol was approved by the institutional review board of National Medical Center (IRB no. H-1811-096-002) and Seoul National University Hospital (H-2006-090-1132).

## 3. Results

### 3.1. Characteristics of the Study Participants

Between 11 May 2017 and 13 January 2019, a total of 180 patients were enrolled at the National Medical Center. There were 65 subjects in the ATB group, 53 in the LTBI group, and 62 in the NI group. The baseline characteristics of the participants are summarized in Table 1. Age and sex were significantly different among the three groups. Age was highest in the ATB group and lowest in the NI group (*p* < 0.0001). The proportion of males in the ATB group was highest (*p* < 0.0001). None of the subjects in the NI and LTBI groups had a previous history of TB.

### 3.2. Serum Levels of Immunoglobulin G (IgG) and Immunoglobulin A (IgA) against Mycobacterium Tuberculosis Chorismate Mutase (TBCM), Antigen 85B (Ag85B), Early Secreted Antigen-6 (ESAT-6), and Culture Filtrate Protein-10 (CFP-10)

**Figure 1:**
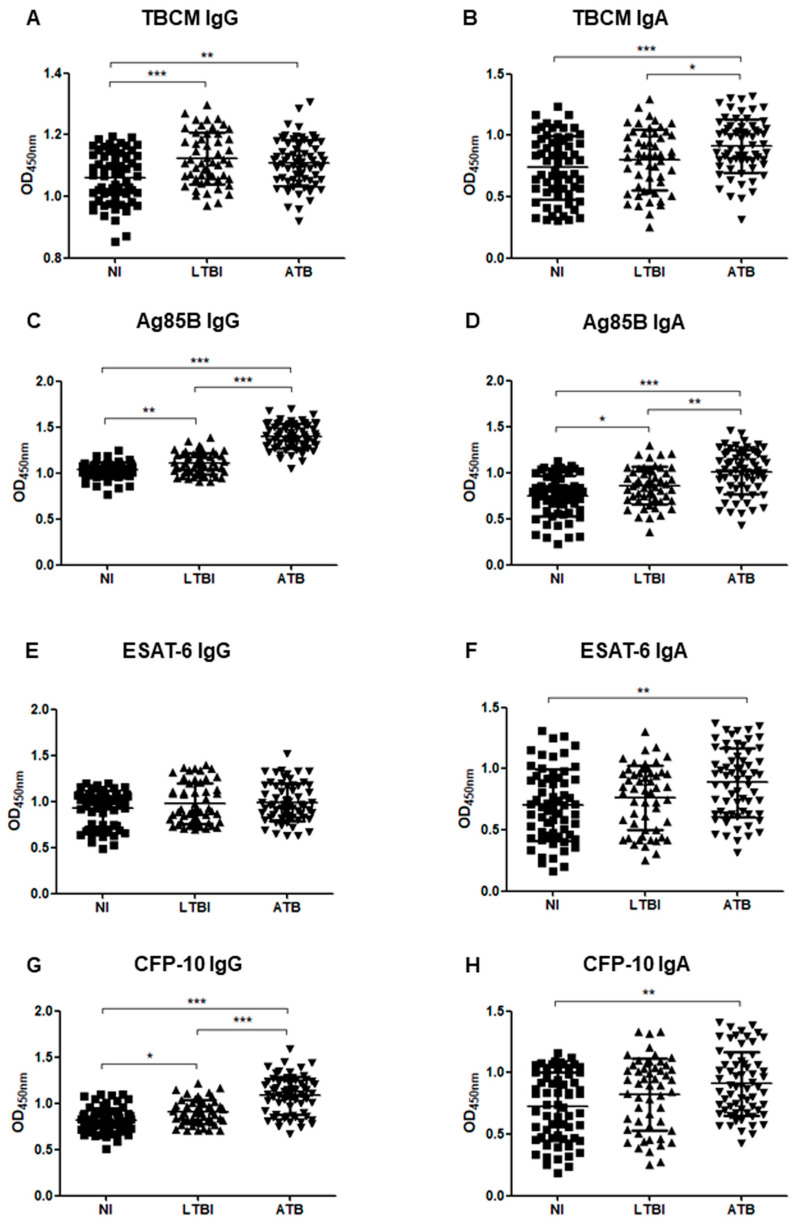
The optical density (OD) values of the anti-TBCM (*Mycobacterium tuberculosis* chorismate mutase) immunoglobulin G (IgG) (**A**), anti-TBCM immunoglobulin A (IgA) (**B**), anti-antigen 85B (Ag85B) IgG (**C**), anti-Ag85B IgA (**D**), anti-ESAT-6 (early secreted antigen-6) IgG (**E**), anti-ESAT-6 IgA (**F**), anti-CFP-10 (culture filtrate protein-10) IgG (**G**), and anti-CFP-10 IgA (**H**) serodiagnosis assays for the comparison of 65 active tuberculosis (ATB), 53 latent tuberculosis infection (LTBI), and 62 non-infected individuals (NI). Each dot represents the values obtained from individual subjects, and horizontal bars indicate the average values. Groups were compared by one-way analysis of variance (ANOVA) with Bonferroni’s multiple comparisons. * *p* < 0.05; ** *p* < 0.01; *** *p* < 0.001.

Figure 1 shows the IgG and IgA responses to TBCM, Ag85B, ESAT-6, and CFP-10 in each participant group.

IgG levels against TBCM were significantly higher in LTBI (*p* < 0.0001) and ATB groups (*p* = 0.004) than in the NI group. No significant difference was found between LTBI and ATB individuals (Figure 1A). IgA levels against TBCM were significantly higher in the ATB group than in the NI (*p* < 0.0001) and LTBI groups (*p* = 0.047). No significant difference was found between the NI and LTBI groups (Figure 1B).

The levels of both IgG and IgA against Ag85B were significantly higher in the LTBI (*p* = 0.005, *p* = 0.022, respectively) and ATB (*p* < 0.0001 in both cases) groups than the NI group, as well as in the ATB group compared to those in the LTBI group (*p* < 0.0001, *p* = 0.002, respectively; Figure 1C,D). The levels of IgG against ESAT-6 were not significantly different among NI, LTBI, and ATB groups (Figure 1E). The levels of IgA against ESAT-6 were significantly higher in the ATB group than in the NI group (*p* = 0.001). There was no significant difference in levels between the NI and LTBI groups and between the LTBI and ATB groups (Figure 1F).

The IgG levels against CFP-10 were significantly higher in LTBI (*p* = 0.010) and ATB (*p* < 0.001) groups than in the NI group, as well as in the ATB group (*p* < 0.0001) compared to those in the LTBI group (Figure 1G). The IgA levels against CFP-10 were significantly higher in the ATB group than in the NI group (*p* = 0.001). There were no significant differences in CFP-10 IgA levels between the NI and LTBI groups and between the LTBI and ATB groups (Figure 1H).

### 3.3. Serum Levels of IgG and IgA in Subgroup Analysis

**Figure 2:**
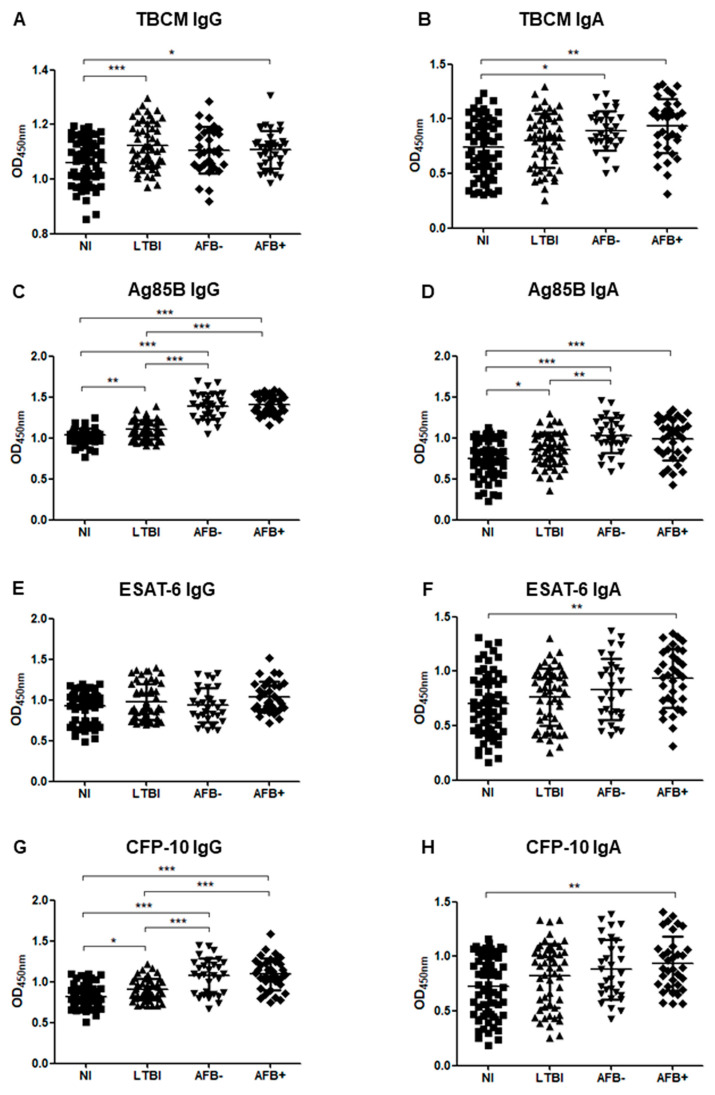
The optical density (OD) values of the anti-TBCM IgG (**A**), anti-TBCM IgA (**B**), anti-Ag85B IgG (**C**), anti-Ag85B IgA (**D**), anti-ESAT-6 IgG (**E**), anti-ESAT-6 IgA (**F**), anti-CFP-10 IgG (**G**), and anti-CFP-10 IgA (**H**) serodiagnosis assays for the comparison of 31 acid-fast bacilli (AFB)-negative tuberculosis (AFB-), 34 AFB-positive tuberculosis (AFB+), 53 latent tuberculosis infection patients (LTBI), and 62 non-infected individuals (NI). Each dot represents the values obtained from individual subjects, and horizontal bars indicate the average values. Groups were compared by one-way ANOVA with Bonferroni’s multiple comparisons. * *p* < 0.05; ** *p* < 0.01; *** *p* < 0.001.

Figure 2 shows the IgG and IgA responses to each antigen when ATB patients were classified into acid-fast bacilli (AFB)-negative and AFB-positive TB groups. The levels of IgG against Ag85B and CFP-10 showed significant differences when comparing subgroups as follows: NI and AFB-negative TB (*p* < 0.001), NI and AFB-positive TB (*p* < 0.001), LTBI and AFB-negative TB (*p* < 0.001), LTBI and AFB-negative TB (*p* < 0.001), and LTBI and AFB-negative TB (*p* < 0.001); levels increased from NI to LTBI to AFB-negative and finally AFB-positive TB (Figure 2C,G). The IgA levels against ESAT-6 and CFP-10 were significantly higher in the AFB-positive TB group than in the NI group, but there was no significant difference between the NI and AFB-negative TB groups (Figure 2F,H).

**Figure 3:**
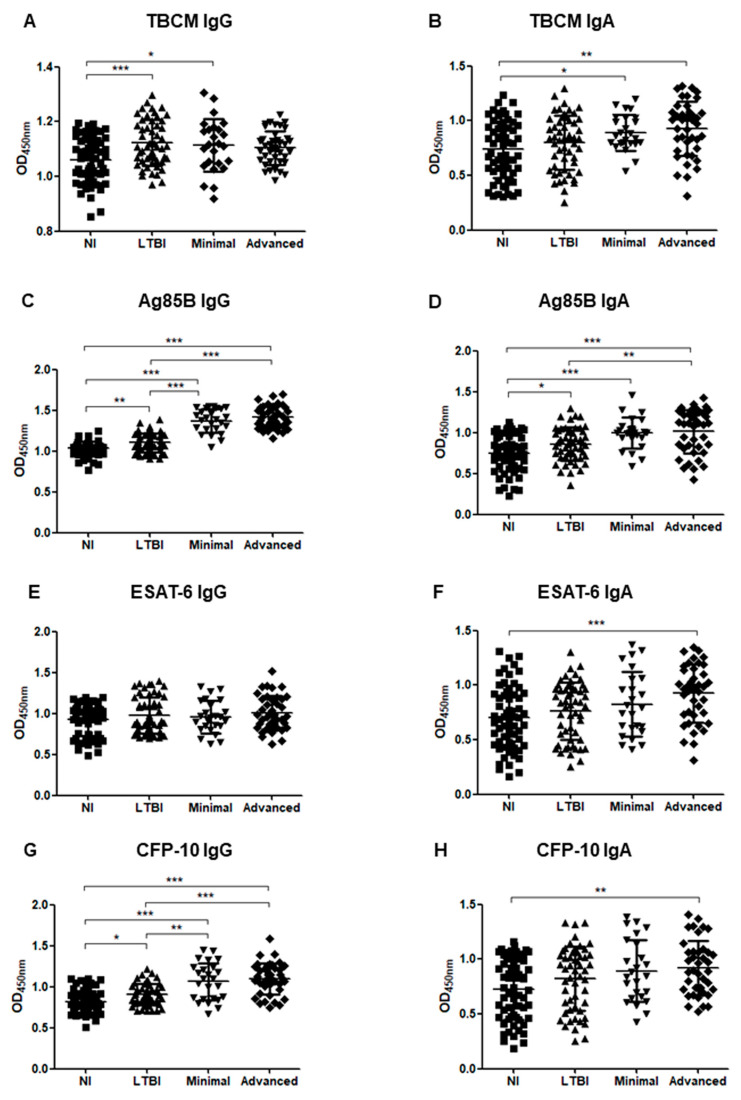
The optical density (OD) values of the anti-TBCM IgG (**A**), anti-TBCM IgA (**B**), anti-Ag85B IgG (**C**), anti-Ag85B IgA (**D**), anti-ESAT-6 IgG (**E**), anti-ESAT-6 IgA (**F**), anti-CFP-10 IgG (**G**), and anti-CFP-10 IgA (**H**) serodiagnosis assays for the comparison of 25 minimal tuberculosis, 40 advanced tuberculosis, 53 latent tuberculosis infection patients (LTBI), and 62 non-infected individuals (NI). Each dot represents the values obtained from individual subjects, and horizontal bars indicate the average values. Groups were compared by one-way ANOVA with Bonferroni’s multiple comparisons. * *p* < 0.05; ** *p* < 0.01; *** *p* < 0.001.

Similarly, when ATB patients were classified into minimal and advanced TB groups according to their radiological severity, the levels of IgG against Ag85B and CFP-10 showed significant differences between most groups as follows: NI and minimal (*p* < 0.001), NI and advanced (*p* < 0.001), LTBI and minimal (*p* < 0.001), LTBI and advanced (*p* < 0.001); the values tended to increase from NI, LTBI, minimal, and finally to advanced TB (Figure 3C,G). The levels of IgA against Ag85B were also significantly higher in the minimal TB group (*p* < 0.001) and the advanced TB group (*p* < 0.001) than in the NI group and were significantly higher in the advanced TB group than in the LTBI group (*p* = 0.008), but there was no significant difference between the LTBI and minimal TB groups (Figure 3D). The levels of IgA against ESAT-6 and CFP-10 were significantly higher in the advanced TB group than in the NI group, but there was no significant difference between the NI and minimal TB groups (Figure 3F,H).

**Figure 4:**
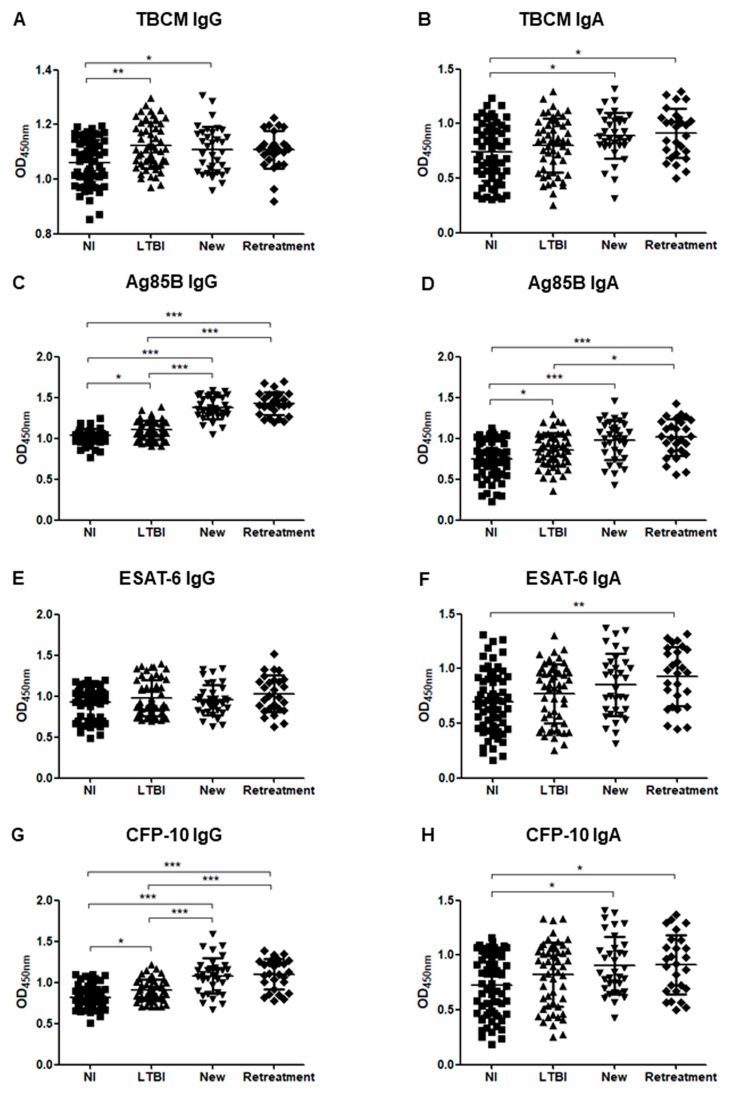
The optical density (OD) values of the anti-TBCM IgG (**A**), anti-TBCM IgA (**B**), anti-Ag85B IgG (**C**), anti-Ag85B IgA (**D**), anti-ESAT-6 IgG (**E**), anti-ESAT-6 IgA (**F**), anti-CFP-10 IgG (**G**), and anti-CFP-10 IgA (**H**) serodiagnosis assays for the comparison of 34 new patients, 27 retreatment patients, 53 latent tuberculosis infection patients (LTBI), and 62 non-infected individuals (NI). Each dot represents the values obtained from individual subjects, and horizontal bars indicate the average values. Groups were compared by one-way ANOVA with Bonferroni’s multiple comparisons. * *p* < 0.05; ** *p* < 0.01; *** *p* < 0.001.

Figure 4 shows the IgG and IgA responses to each antigen when ATB patients were divided into new patients and retreatment groups. For the levels of IgG against Ag85B and CFP-10, no significant difference was observed between the new patient and retreatment groups, but there were significant differences between all other groups as follows: NI and new patient (*p* < 0.001), NI and retreatment (*p* < 0.001), LTBI and new patient (*p* < 0.001), LTBI and retreatment (*p* < 0.001). Furthermore, the antibody levels tended to increase in the following order: NI, LTBI, new patient, and retreatment (Figure 4C,G). IgA levels for TBCM and CFP-10 were significantly higher in new patients (*p* = 0.031) and retreatment (*p* < 0.001) groups than in the NI group. There was no significant difference between the LTBI group and both the new patients and retreatment groups and between the new patients and retreatment groups (Figure 4B,H). There was no significant difference in AFB positivity, radiological severity, and comorbidities between the new patient and the retreatment groups.

### 3.4. Correlations among Serum Levels of IgA, IgG, and Interferon-Gamma (IFN-γ)

**Figure 5:**
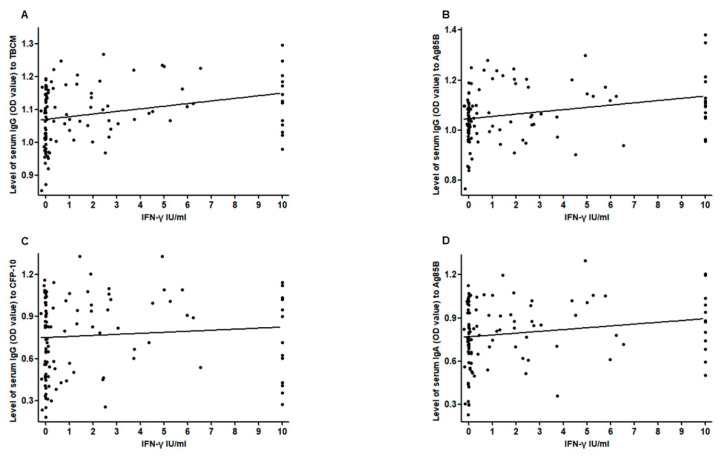
Correlation between the level of interferon-gamma and the level of serum IgG against TBCM (*r* = 0.303, *p* = 0.001) (**A**), IgG against Ag85B (*r* = 0.286, *p* = 0.002) (**B**), IgG against CFP-10 (*r* = 0.259, *p* = 0.005) (**C**), and IgA against Ag85B (*r* = 0.193, *p* = 0.039) (**D**).

There were trends of a positive correlation between the level of IFN-γ induced by the QFT-GIT test and the OD values of serum IgG against Ag85B (r = 0.286, *p* = 0.002), TBCM (r = 0.303, *p* = 0.001), and CFP-10 (r = 0.259, *p* = 0.005) and IgA against Ag85B (r = 0.193, *p* = 0.039; Figure 5). There was a strong positive correlation between the serum levels of IgA against CFP-10 and ESAT-6 in ATB subjects (r = 0.871, *p* < 0.001). There were also positive correlations between the serum levels of IgA against CFP-10 and TBCM (r = 0.786, *p* < 0.001), Ag85B (r = 0.720, *p* < 0.001), and ESAT-6 (r = 0.878, *p* < 0.001) in LTBI subjects. Similarly, there were significant positive correlations between the serum levels of IgG against CFP-10 and Ag85B (r = 0.720, *p* < 0.001) in LTBI subjects.

### 3.5. Diagnostic Performance of Antibodies against Mtb Antigens for Diagnosis of Latent Tuberculosis Infection (LTBI) or Active Tuberculosis (ATB)

**Figure 6:**
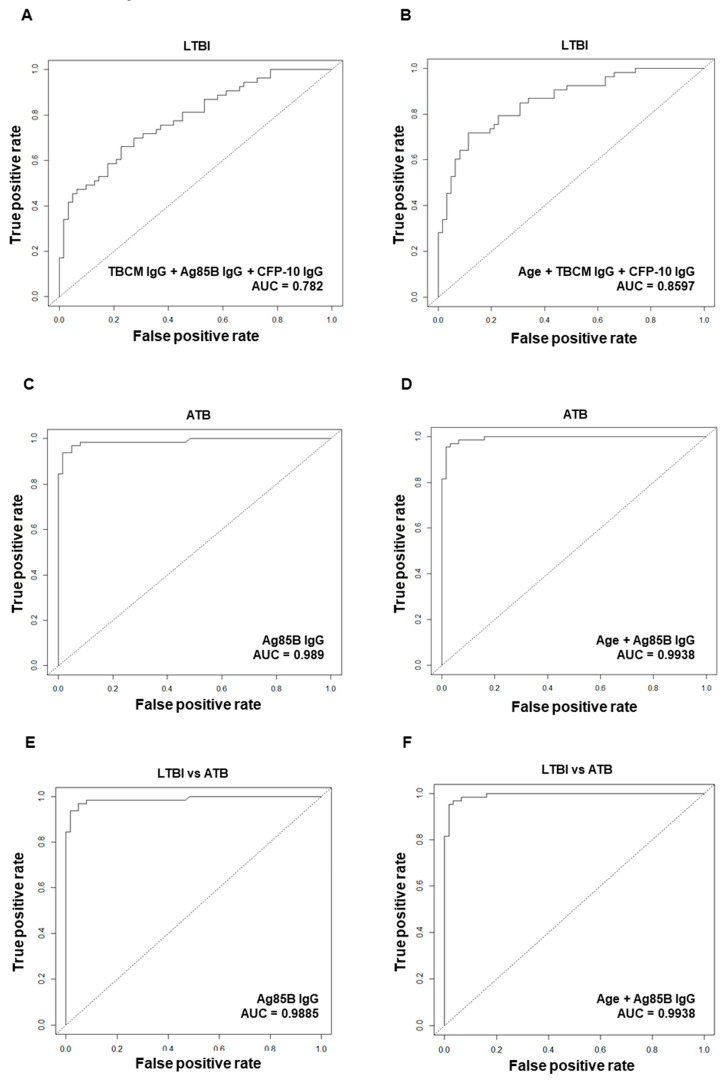
Receiver operating characteristic (ROC) curve analysis and logistic regression models were used to evaluate the discriminating power of biomarkers between non-infected (NI) and latent tuberculosis infection (LTBI) (**A**, **B**), NI and active tuberculosis (ATB) (**C**, **D**), and LTBI and ATB (**E**, **F**). The following biomarkers were used for each model configuration: IgG levels against TBCM, Ag85B, and CFP-10 (**A**); age and IgG levels against TBCM and CFP-10 (**B**); IgG against Ag85B (**C**); age and IgG against Ag85B (**D**); IgG against Ag85B (**E**); age and IgG against Ag85B (**F**).

During the multivariate analysis, we performed logistic regression analysis by including significant variables from the univariable analysis and additionally generated models that included age and sex. As a result of multivariate logistic regression analysis to investigate independent variables significantly related to LTBI diagnosis, the IgG levels against TBCM, Ag85B, and CFP-10 were independently related to LTBI diagnosis. ROC curve analysis was performed for LTBI diagnostic performance, and the AUC combining each antibody value was highest with a value of 0.782 (Figure 6A), compared to the AUC value of each IgG against TBCM, Ag85B, and CFP-10. When adjusting for age and sex, IgG against TBCM and CFP-10 and age were independently associated with LTBI diagnosis. When the ROC analysis was performed including these markers, the AUC value was 0.8597 (Figure 6B), which increased the predictive power compared to the model without age.

In the multivariate analysis related to ATB diagnosis, the IgG level against Ag85B was independently significantly related to the diagnosis of ATB, and the ROC analysis showed a strong diagnostic performance of 0.989 (Figure 6C). When age and sex were adjusted, IgG against Ag85B and age were independently significant factors, and in this model, the AUC was 0.9938 (Figure 6D), showing increased predictive power compared to the model without age. In the multivariate analysis of the differential diagnosis of LTBI and ATB, only IgG against Ag85B was an independently significant marker, showing great differentiating power (AUC: 0.9885) in the ROC analysis (Figure 6E). When adjusting for age and sex, IgG against Ag85B and age were two independently significant variables. In this model, the AUC was 0.9938 (Figure 6F), with higher predictive power than the model without age.

**Figure 7:**
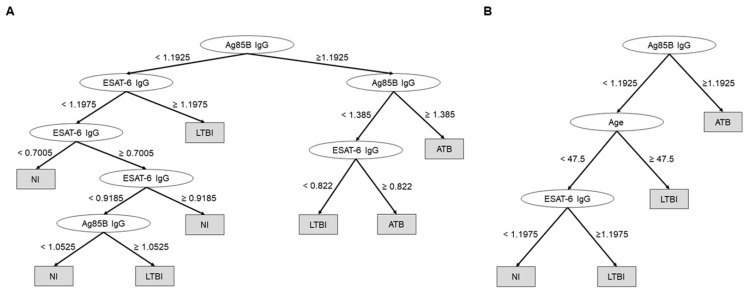
Decision-tree models to comprehensively predict the diagnosis of active tuberculosis (ATB) or latent tuberculosis infection (LTBI), using IgG against Ag85B and ESAT-6 (accuracy = 0.789) (**A**) and using age and IgG against Ag85B and ESAT-6 (accuracy = 0.8269) (**B**).

Additionally, we created a decision-tree model using IgG against Ag85B and ESAT-6 to predict comprehensively the diagnosis of ATB or LTBI. The accuracy had a significant diagnostic value of 0.789 (Figure 7A). Moreover, when age was included in the model, this value was improved to 0.8269 (Figure 7B).

## 4. Discussion

In the present study, we compared IgG and IgA responses to TB antigens TBCM, Ag85B, ESAT-6, and CFP-10 in groups of NI, LTBI, and ATB subjects. The levels of IgG and IgA against Ag85B and IgG against CFP-10 were significantly higher in ATB patients, followed by LTBI patients, and were lowest in NI subjects. The IgG against TBCM was significantly higher in LTBI subjects than in NI subjects. When age and sex were adjusted in the multivariate analysis, IgG against TBCM and CFP-10 were independently related to LTBI diagnosis. Additionally, IgG against Ag85B was a significant marker, independently related to both ATB diagnosis and discrimination between ATB and LTBI.

Our results suggest that levels of IgA and IgG correlate with bacillary load, which is consistent with the results of previous studies. Abebe et al. reported that the levels of IgA and IgG for Rv2031 were significantly higher in untreated TB patients, the next highest in generation contactors, and the lowest in the control cohort. This shows that the levels of IgA and IgG are strongly correlated with bacillary load [20]. Similarly, Kunnath-Velayudhan et al. reported that antibody levels were correlated with bacterial load when patients with suspected TB were classified according to sputum smear and bacterial load [9]. When the burden of the bacilli is low, such as in LTBI or minimal TB, the concentration of membrane-associated proteins derived from the bacilli is low. However, when the burden of the bacilli increases, such as in severe ATB, the activated bacilli secrete proteins, which is accompanied by an increase in antibody responses. Thus, these antibody response titers might reflect the status of TB disease or the progression of infection. In our study, the levels of IgG against Ag85B and CFP-10 could significantly discriminate specific TB subgroups, even when the ATB group was classified based on radiological severity, AFB smear results, and past treatment history. These results suggest that the antibody responses to specific TB antigens have diagnostic potential for use in discriminating the detailed spectrum of TB associated with bacillary load.

IgG against TBCM, a novel antigen, was an independent significant marker for LTBI diagnosis. The diagnostic model including IgG against TBCM in both models with and without age showed good predictability. In addition, there were positive correlation trends between the level of IFN-γ induced by the QFT-GIT test and the serum level of IgG against TBCM in LTBI and NI subjects. These results suggest that IgG against TBCM could potentially complement current diagnostic methods in the diagnosis of LTBI. Previous studies have shown that TBCM is active in acidic environments, such as infected macrophages, and is essential for TB survival by regulating intermediates that are important for the biosynthesis of a wide range of compounds [21]. As *Mtb* could be starved due to nutrient deficiency inside macrophages in latent stages [22,23], the metabolism of *Mtb* in LTBI might have a significant relationship with survival. In addition, since TB could present different gene expression patterns depending on the stage of infection, differences in immune responses to stage-specific antigens might occur [24]. Our result suggests that TBCM could be associated with the survival of *Mtb* in the latent stage of the disease.

In the present study, levels of IgG against Ag85B and CFP-10 were significantly different between LTBI and AFB-negative- or minimal TB, showing excellent discriminatory power between the detailed spectrum of TB. In particular, Ag85B IgG was not only highly predictive in the ATB diagnosis and discrimination between LTBI and ATB, regardless of age in the multivariate analysis diagnostic model, but also a significant factor in the decision tree model. Therefore, our results can be used to compensate for limitations of the current LTBI diagnosis methods that cannot distinguish between LTBI and ATB. The role of antigens is important in serological diagnostic methods using ELISA. It is necessary for the TB-specific antigen to induce a meaningful immune response, and there should be minimal cross-reactivity to increase sensitivity and specificity. As the antigenic components of TB are complex and there are cross reactions with other species, efforts to isolate and assay antigenic components specific for TB are necessary. Early antigens were mixtures of constituents of *Mtb*, such as purified protein derivatives and, hence, many new antigens, including A60, Ag85, 38 kDa, 16 kDa, MPT51, *Mtb*48, ESAT-6, CFP-10, 2,3-diacyltrehalose (DAT), 2,3,6-triacyltrehalose (TAT), and lipoarabinomannan (LAM), have been developed to increase sensitivity and specificity [25]. However, the accuracy of serodiagnosis in TB varies widely between studies, and the sensitivity of each study ranges from 0% to 100%, with specificities from 31% to 100% [26,27]. Previous studies using Ag85B, ESAT-6, and CFP-10 also showed varying results in terms of diagnostic accuracy. Kumar et al. reported that the sensitivity of IgG against Ag85 in the diagnosis of ATB was higher (84.1%) than that of IgG to CFP-10 (66%) and ESAT-6 (64.9%) [28]. In another study, the sensitivity of IgG against Ag85 in the diagnosis of ATB was 67.5%, higher than that of IgA and immunoglobulin M (IgM) against Ag85 [29]. However, another study reported the highest AUC of IgG against ESAT-6 when evaluating the value of IgG against Ag85B, ESAT-6, and CFP-10 in the diagnosis of ATB [30]. The results of our study showed higher diagnostic accuracy than that of previous studies using similar antigens. These differences might be attributed to the purity of the antigens or differences in the study populations. In contrast to the absence or low number of LTBI comparisons in other studies, the present study included a relatively large number of LTBI patients, diagnosed with the QFT-GIT assay. In addition, we confirmed that the QFT-GIT assay results were negative for the healthy control group.

Previous studies have used immunoglobulin isotopes to highlight differences in immune responses. In several studies, IgG has been shown to have useful value in the serodiagnosis of TB and has been reported to be more sensitive than IgA or IgM [31,32,33,34,35]. IgA is relatively less established, but some studies have reported that IgA has higher sensitivity or specificity than IgG and is of diagnostic importance [8,35,36]. *Mtb* invades the respiratory tract and multiplies in it, and it is feasible that the production of IgA could be stimulated during the contact between TB antigens and the mucosal surface [37]. However, studies have shown that IgM is associated with non-specific reactions and is not correlated with the severity of TB [35,38]. IgM is mainly produced in the early stages of infection. Therefore, the value of IgM in the diagnosis of TB might be relatively low because IgG, rather than IgM, increases as time passes after infection or when TB is reactivated [39,40]. However, another study reported that IgG and IgM are both elevated during infection, which indicates conflicting results [41]. Similar to previous studies, the antibody responses to different antigens in our study varied by isotype, and the IgG response appeared to be more discriminate than IgA.

The present study has several limitations. When the ATB group was divided into subgroups, the number of samples in each subgroup was reduced, and this was insufficient to prove statistical significance among the subgroups. There were also differences in age and sex between NI, LTBI, and ATB groups. We adjusted each variable through multivariate analysis. However, if a larger number of samples were obtained, the diagnostic usefulness of each marker in the age- and sex-matched comparison groups could be confirmed.

In conclusion, results of the present study showed that IgG antibody responses to TBCM, Ag85B, and CFP-10 show potential in diagnosing TB. Further, these responses could significantly discriminate between NI, LTBI, and ATB individuals. These results suggest that TB antigen-specific antibodies could be used to develop reliable ELISA tests for the diagnosis of TB.

## Figures and Tables

**Table 1:** Demographic and clinical characteristics of the study population.

	NI (*n* = 62)	LTBI (*n* = 53)	ATB (*n* = 65)
**Demographics**			
Age (years), median (IQR)	33 (29–33)	49 (33–57)	60 (51–67)
Sex			
Male	21 (33.9%)	21 (39.6%)	56 (86.2%)
Female	41 (66.1%)	32 (60.4%)	9 (13.8%)
Body-mass index (kg/m^2^), median (IQR)	22.4 (20.2–24.9)	23.7 (21.4–26.7)	20 (17.0–21.7)
Comorbidities			
Diabetes	3 (4.8%)	6 (11.3%)	14 (21.5%)
Chronic alcoholics	0 (0%)	1 (1.9%)	9 (13.8%)
Cancer	0 (0%)	5 (9.4%)	4 (6.2%)
Liver disease	0 (0%)	4 (7.5%)	7 (10.8%)
Chronic kidney disease	0 (0%)	2 (3.8%)	2 (3.1%)
Heart disease	0 (0%)	3 (5.7%)	3 (4.6%)
Previous history of TB treatment	n/a	n/a	
New patients			34 (52.3%)
Retreatment			27 (41.5%)
Unknown previous TB treatment history			4 (6.2%)
**Bacteriological examinations**			
Acid-fast staining of sputum	n/a	n/a	
Negative			31 (47.7%)
1+			16 (24.6%)
2+			7 (10.8%)
3+			5 (7.7%)
4+			6 (9.2%)
Positive *Mtb* culture of sputum	n/a	n/a	
Liquid media			46 (70.8%)
Solid media			39 (60.0%)
Positive Xpert MTB/RIF assay	n/a	n/a	59 (90.8%)
Drug resistance	n/a	n/a	14 (21.5%)
**Radiographical examinations**			
Normal	61 (98.4%)	51 (96.2%)	n/a
Previously healed TB *	1 (1.6%)	2 (3.8%)	n/a
Radiologic severity of ATB	n/a	n/a	
Minimal			25 (38.5%)
Advanced			40 (61.5%)
Presence of cavity	n/a	n/a	31 (47.7%)

IQR: interquartile range; NI, non-infected; LTBI, latent tuberculosis infection; ATB, active tuberculosis; TB, tuberculosis; Mtb, *Mycobacterium tuberculosis*; n/a, not applicable. * Radiographic lesions suggesting TB sequelae without clinical or microbiological evidence of active pulmonary TB.
